# Novel insights into vascularization patterns and angiogenic factors in glioblastoma subclasses

**DOI:** 10.1007/s11060-016-2269-8

**Published:** 2016-09-15

**Authors:** Siobhan Conroy, Michiel Wagemakers, Annemiek M. E. Walenkamp, Frank A. E. Kruyt, Wilfred F. A. den Dunnen

**Affiliations:** 1 Department of Pathology and Medical Biology (Division of Pathology), University of Groningen, University Medical Center Groningen, HPC EA10, P.O. Box 30.001, 9700 RB Groningen, The Netherlands; 2 Department of Neurosurgery, University of Goningen, University Medical Center Groningen, Groningen, The Netherlands; 3 Department of Medical Oncology, University of Groningen, University Medical Center Groningen, Groningen, The Netherlands

**Keywords:** Angiogenesis, Glioblastoma, Subclasses, Subtypes

## Abstract

Glioblastoma (GBM) is a highly vascularized and aggressive type of primary brain tumor in adults with dismal survival. Molecular subtypes of GBM have been identified that are related to clinical outcome and response to therapy. Although the mesenchymal type has been ascribed higher angiogenic activity, extensive characterization of the vascular component in GBM subtypes has not been performed. Therefore, we aimed to investigate the differential vascular status and angiogenic signaling levels in molecular subtypes. GBM tissue samples representing proneural *IDH1* mutant, classical-like and mesenchymal-like subtypes were analyzed by morphometry for the number of vessels, vessel size and vessel maturity. Also the expression levels of factors from multiple angiogenic signaling pathways were determined. We found that necrotic and hypoxic areas were relatively larger in mesenchymal-like tumors and these tumors also had larger vessels. However, the number of vessels, basement membrane deposition and pericyte coverage did not vary between the subtypes. Regarding signaling patterns the majority of factors were expressed at similar levels in the subtypes, and only ANGPT2, MMP2, TIMP1, VEGFA and MMP9/TIMP2 were higher expressed in GBMs of the classical-like subtype. In conclusion, although morphological differences were observed between the subtypes, the angiogenic signaling status of GBM subtypes seemed to be rather similar. These results challenge the concept of mesenchymal GBMs being more angiogenic than other subclasses.

**Electronic supplementary material:**

The online version of this article (doi:10.1007/s11060-016-2269-8) contains supplementary material, which is available to authorized users.

## Introduction

Glioblastoma (GBM) is the most common primary brain tumor and is among the most vascularized solid tumors [1–4]. The dismal prognosis of patients with GBM warrants development of more effective therapies. Despite the general consensus that GBM comprises a tumor with extensive heterogeneity, all patients are currently still treated uniformly [2, 5, 6].

Molecular subclasses have been described over the past decade based on gene expression patterns, but the discussion on the exact number of subclasses is still ongoing. Initially a set of three subclasses was identified [7], but the definition was strongly refined, expanded to four subclasses and specific molecular aberrations were coupled to the subclasses by The Cancer Genome Atlas (TCGA) Network [8]. The discussion is still not definite and only recently another proposition for subgroups of gliomas has been postulated [9]. From the earlier profiling endeavors, however, three molecular subclasses can be distilled consistently which include the proneural (PN), classical (CLAS) and mesenchymal (MES) subclasses.

Thus far only few studies have reported on the angiogenic properties of the molecular subclasses. Platelet/endothelial cell adhesion molecule 1 (PECAM1), vascular endothelial growth factor A (VEGFA) and vascular endothelial growth factor receptor 1 and 2 (VEGFR1, 2) were reported to be upregulated in MES GBMs [7]. Other results only have described differential responses of GBMs to anti-angiogenic treatment in both the pre-clinical and clinical setting. One study has addressed whether the differences in response were associated with the molecular subclasses and reported selected benefit for PN subclass [10]. In an aortic ring assay anti-VEGFA treatment abrogated microvessel sprouting in one cell line but not in the other [11], and tumor recurrences following anti-VEGFA treatment could be divided in distinct resistance phenotypes [12].

Since the subclasses are characterized by molecular aberrations, differential employment of angiogenic signaling pathways could also be suggested based on the defining features of the subclasses. It has for example been shown that IDH1^mutant^ protein, which is exclusively expressed in PN GBMs, stabilizes HIF-1α-expression [13]. HIF-1α on its turn is known as a potent inducer of angiogenesis via multiple signaling pathways [14]. The expression of EGFRvIII, the constitutively active (mutant) form of *EGFR* characteristic of CLAS GBMs, was on the other hand reported to promote angiogenesis by activating the IL-8 pathway and inducing expression of cytokines and interleukins [15, 16].

To determine whether specific angiogenesis-related factors could serve as possible new candidates for GBM subclass-differentiated anti-angiogenic therapy, we assessed the vascular status of the subclasses using morphometry, immunohistochemistry (IHC) and polymerase chain reaction (PCR).

## Methods

### Patient population

Tissue samples from 30 patients diagnosed with GBM were selected for the Groningen cohort. All patients underwent neurosurgical debulking at the University Medical Center Groningen (UMCG) in the Netherlands in the period August 2006 to May 2012. The mean age at diagnosis was 55 and the higher incidence rate in males was reflected in the male to female ratio (67 % male) [17]. The samples selected for this study, 10 per subclass, were all previously identified as PN (*IDH1* ^*mut*^), CLAS-like and MES-like subclasses [18]. Due to the fact that this cohort of GBMs was not subclassified transcriptionally but instead through a protein-based approach, we refer to the subclasses of these tumors as PN *IDH1* ^*mut*^, CLAS-like and MES-like where the analyses concern these tumors. Archival tissue of all patients was handled according to the Dutch Code of Conduct for proper secondary use of human tissue (http://www.federa.org).

Level 3 gene expression data was obtained of 146 GBM patients that were previously transcriptionally subclassified (core TCGA samples, http://cancergenome.nih.gov/) [8]. The age at diagnosis in this cohort was 55 and the male percentage was 62. Clinical characteristics of both cohorts are summarized in Table 1.

**Table 1:** Summary of characteristics of patients with GBM in the Groningen and Verhaak cohort

Characteristic	Groningen cohort	Verhaak cohort
Number of patients	30	146
Mean age at diagnosis (95 % CI)	55 (49–60)	55 (53–58)
Median overall survival in days (range)	526 (62–1447)	361 (0–3524)
Male sex (%)	20 (67)	91 (62)
Female sex (%)	10 (33)	55 (38)

### Necrosis measurement on MRI scans

Pre-operative imaging of the Groningen cohort was used to measure the volume of central necrosis in relation to total tumor volume. Volume contrast-enhanced T1 MRIs suitable for volume measurements were available for 24 of 30 patients. Amongst the 6 patients lacking a scan of sufficient quality, there were 3 PN *IDH1* ^*mut*^, 2 CLAS-like and 1 MES-like tumor. The scans were analyzed three-dimensionally in Brainlab iPlan® Cranial planning software version 3.0 (Brainlab AG, Feldkirchen, Germany), on which the enhanced area was interpreted as vital tumor, and the non-enhanced central area of the tumor as necrosis. As a measurement for total tumor area the enhanced and the non-enhanced central area were added up.

### IHC procedure

Sections were cut from FFPE tissue in series and IHC staining was performed as described previously [18]. Sections were stained for Carbonic anhydrase IX (CAIX), CD34, Endoglin (ENG), collagen type IV alpha 1 (ColIV) and α-smooth muscle actin (α-SMA).

### Morphometrical analyses

The microvascular density (MVD) was microscopically assessed using the Chalkley grid. The average number of vessels per mm^2^, average vessel area and vessel perimeter were determined using computer-assisted morphometry.

### Histological evaluation

The expression levels of CAIX, CD34, ENG, ColIV and α-SMA were quantified using Aperio ImageScope software. Vital tumor tissue was delineated on every individual section and staining, and hypoxic tissue was delineated using the CAIX staining pattern as guidance. The positive pixel percentage was calculated by division of the surface area found positive (in pixels) by the total area of the vital, normoxic or hypoxic field (in pixels), a method previously applied by others [19, 20]. Since staining for α-SMA and ColIV was specifically identified around vessels, an indication of the thickness of these layers was obtained by dividing the number of positive pixels by the total vessel perimeter of that sample.

### Microfluidic cards

RNA was purified from 30 snap-frozen GBM biopsy samples and reverse transcribed to cDNA. Custom-designed Taqman array Micro Fluidic Cards (low-density array, Applied Biosystems, Foster City, CA) were used to obtain gene expression analyses of these samples by analysis on a ViiA™ 7 real-time sequence detection system (Applied Biosystems). Assays were included for 31 genes of interest and one endogenous control.

### Statistical analysis

Statistical analyses were performed using SPSS software version 22.0 (SPSS, Chicago, IL) and visualized using Graphpad Prism version 5 (Graphpad Software Inc, San Diego, CA). Correlations were calculated by Spearman’s rho (SR). Normal distributions were tested by one-way ANOVA or *t* test and data not normally distributed was tested by a Kruskal–Wallis test or Mann–Whitney U test. The multiple group comparisons were followed up by either Tukey’s or Dunn’s post-hoc tests. *P* values < 0.05 were considered significant and in all cases exact two-sided *P* values were reported.

Supplementary Methods can be found online (Online Resource 1).

## Results

### PN *IDH1*^*mut*^ tumors have a better prognosis than MES-like tumors

The probability of overall survival (OS) was assessed to determine whether IHC-based molecular stratification identified subclasses with similar survival periods as those that were initially reported for the transcription-based molecular subclasses [7, 8]. Kaplan–Meier analysis revealed that the survival of PN *IDH1* ^*mut*^, CLAS-like and MES-like subclasses differed (*P* < 0.05, Log-rank test, Online Resource 2) similar to the originally identified molecular subclasses [7, 8].

### Hypoxic and necrotic tissue areas are more prevalent in MES-like GBMs

The MES subclass has been considered the most angiogenic subclass that also displays a higher level of necrosis [21]. Since hypoxia is known to be a potent inducer of neo-angiogenesis and areas of hypoxia often surround necrotic tissue [22, 23], we determined the size of necrotic and hypoxic tissue on pre-operative MRIs and in biopsied tissue specimens, respectively.

**Fig. 1:**
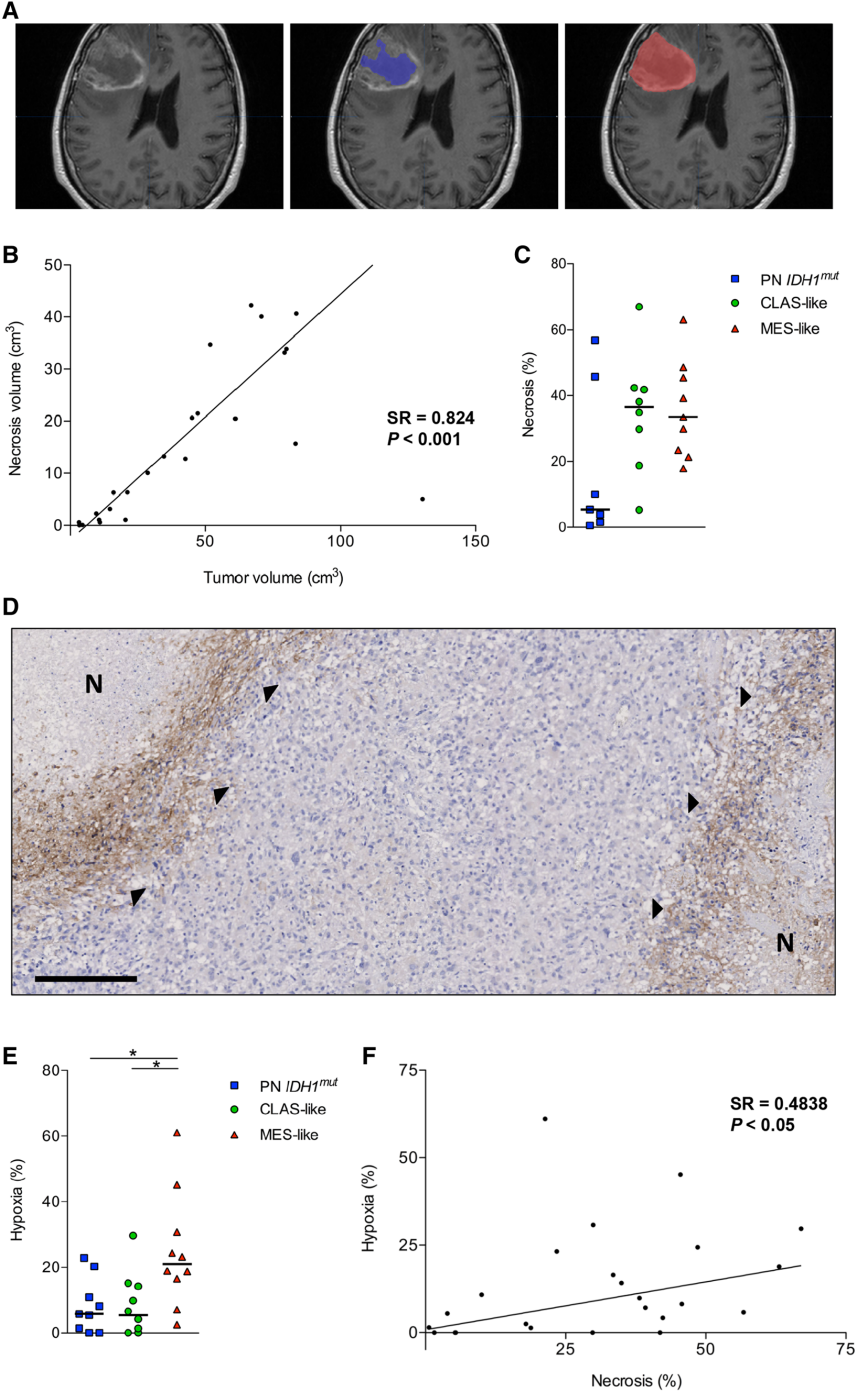
The rate of necrosis is associated with total tumor volume and MES-like tumors have a relatively higher percentage of hypoxic tumor area. Pre-operative radiographic scans of patients were analyzed for the volume of necrosis and total tumor, in which the total tumor area (*right* image) and the central necrosis (*middle* image) were delineated (**a**). The volume of necrosis was found to gradually increase with total tumor volume (SR = 0.824, *P* < 0.01) (**b**), but the percentage of necrosis only showed a trend for a lower level of necrosis in PN *IDH1* ^*mut*^ GBMs (**c**). A representative micrograph of CAIX staining is shown (**d**), where ‘N’ indicates necrotic tissue, and the arrowheads indicate staining for CAIX. A rim surrounding the necrosis is depicted that was positive for CAIX expression. Quantification of these positive CAIX sites and comparing them against total vital tumor area indicated that the percentage of hypoxic tissue is higher in MES-like GBMs compared to CLAS-like GBMs (**e**). The percentage of hypoxic tumor area was found to be associated with the proportion of necrosis measured on the MRIs (SR = 0.4838, *P* < 0.05) (**f**). *Horizontal lines* represent median score of the groups; *scale bar* 250 µm; *P < 0.05

The volume of central necrosis and total tumor volume were calculated in 3D, for which central non-enhanced areas were interpreted as necrosis and volumetrically compared to the contrast-enhanced total tumor size (Fig. 1a). These measurements pointed out that the volume of necrotic tissue was correlated with total tumor volume (SR = 0.824, *P* < 0.01, Fig. 1b), illustrating that larger tumors tend to have more necrosis. Since PN *IDH1* ^*mut*^ tumors appeared smaller on the scans (but not significantly), the level of necrosis needed to be corrected for total tumor volume. The percentage of necrosis, when expressed against the total tumor volume, still showed a trend for a lower level of necrosis in PN *IDH1* ^*mut*^ tumors (Fig. 1c).

The level of hypoxia was assessed through CAIX on tissue sections, a marker known to be suitable for the identification of tumor hypoxia [24]. Staining for CAIX was observed in only a fraction of the vital tissue area and could indeed be observed around necrosis (Fig. 1d). The staining for CAIX was significantly enriched in the tissue areas that were selected as hypoxic (all *P* values < 0.001, Online Resource 3). Of the GBM subclasses the MES-like group appeared to have relatively the largest hypoxic area (Fig. 1e).

In order to connect the necrosis and hypoxia measurements the correlation was determined, which identified an association between the parameters (SR = 0.4838, *P* < 0.05, Fig. 1f). Although the association is already significant as it is, it is conceivable that it would be stronger if the percentage of hypoxic tissue would be a little higher. It is very likely that the level of hypoxia in our analyses did underestimate the true level of hypoxia, as our tissue samples were obtained from vital tumor areas to be most suitable for diagnostic procedures.

### Endothelial marker expression and MVD in GBM subclasses

**Fig. 2:**
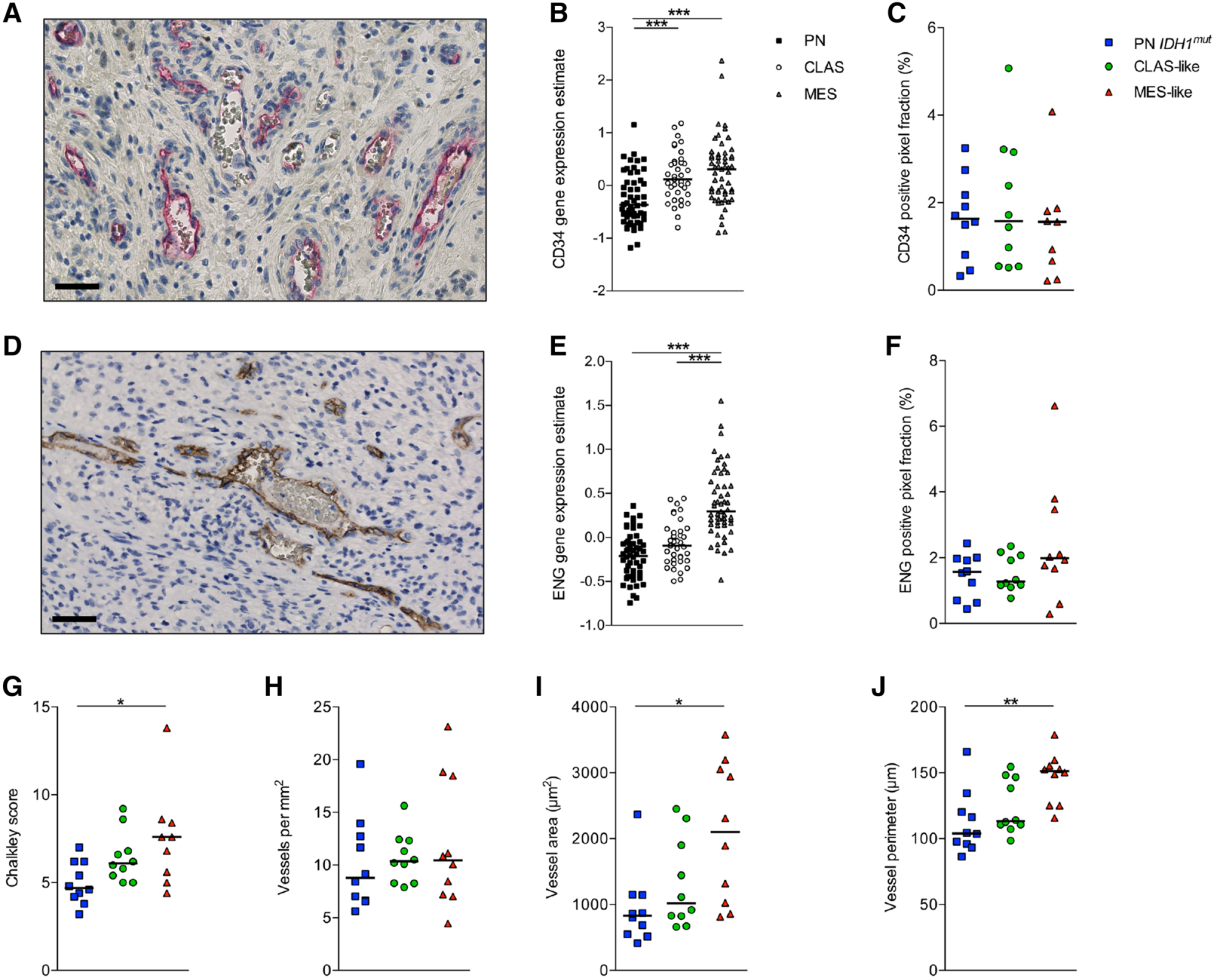
Endothelial marker expression is increased in MES-like GBMs that on average have larger vessels. The expression patterns of endothelial marker CD34 (**a**) and neo-endothelial marker ENG (**d**) is lowest in PN *IDH1* ^*mut*^ tumors and highest in MES tumors at mRNA level (**b, e**). When this is evaluated at protein level, the differences between the subclasses do not maintain for CD34 expression (**c**), but a trend can still be observed for elevated expression of ENG in MES-like tumors (**f**). Morphometric assessments of vessels, including Chalkley grid score (**a**), average vessel area (**c**), and vessel perimeter (**d**) showed that MES-like GBMs have larger vessels than PN *IDH1* ^*mut*^ GBMs, but the number of vessels per mm^2^ (**b**) did not differ. *Horizontal lines* represent median values; *scale bar* 50 µm; *P < 0.05, **P < 0.01, ***P < 0.001

Then the expression of endothelial markers CD34 (Fig. 2a) and neo-endothelial marker ENG (Fig. 2d) was assessed in order to analyze the vascularization patterns. The mRNA expression level in the Verhaak cohort indicated that CD34 and ENG are upregulated in MES tumors in comparison to PN tumors (Fig. 2b, e). At protein level the relative expression of CD34 was however not different between the molecular subclasses (Fig. 2c). Similarly, no differences in relative protein expression of ENG were observed either (Fig. 2f). Using the staining pattern for CAIX as guidance, we then assessed whether the expression differed between normoxic and hypoxic tissue areas. Also in this subdivision of the tissue the expression of endothelial markers remained similar in all subclasses (Online Resource 4).

The vascularization pattern analysis was then continued by the assessment of multiple MVD-parameters. A Chalkley grid evaluation pointed out that MES-like tumors have larger vessels than PN *IDH1* ^*mut*^ tumors (Fig. 2g, P < 0.05). The average vessel area and vessel perimeter were also found to be larger in MES-like tumors (Fig. 2i, j, P < 0.01), but the number of vessels was not different between the subclasses (Fig. 2h). Between normoxic and hypoxic tissue areas only CLAS-like tumors were found to have more vessels in the hypoxic areas (*P* < 0.001), and no differences were observed for the other MVD-parameters (Online Resource 4).

### Vessels of molecular subclasses are not different in maturation status

**Fig. 3:**
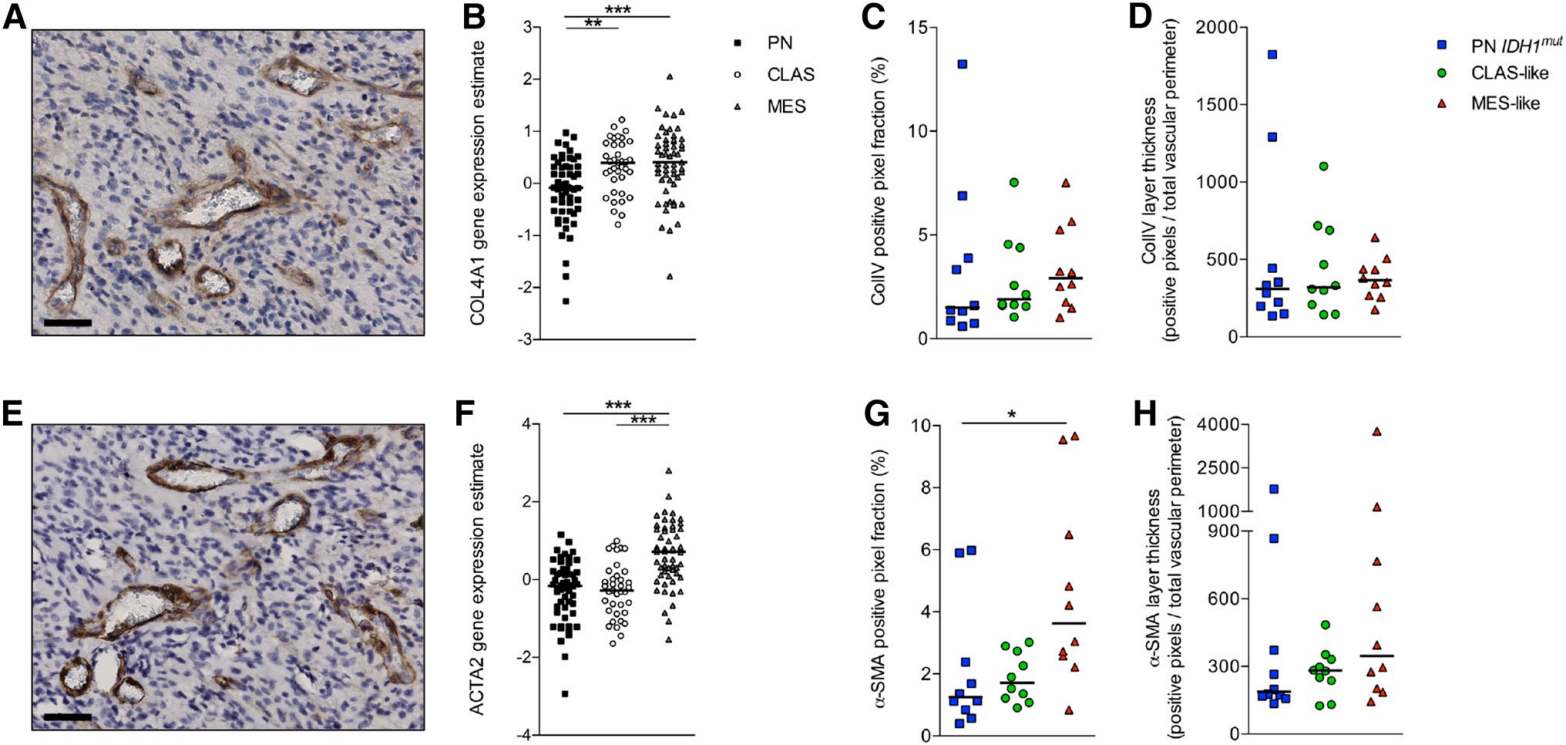
The vascular maturation status of GBM subclasses is rather similar. The mRNA expression of basement membrane marker ColIV (**a**) is highest in CLAS and MES GBMs (**b**). At protein level the expression of ColIV still shows a trend for higher expression in MES-like GBMs when corrected for total tissue area (**c**), but this trend disappears when the layer thickness of ColIV is determined through division of the positive pixels by the total vascular perimeter (**d**). For α-SMA (**e**) an increased mRNA expression level was also observed for MES tumors (**f**), but this remained a significant difference at protein level when we corrected for total tissue area (**g**). The correction for the total vascular perimeter eventually reduced the differences in α-SMA expression to a trend for elevated expression in MES-like tumors (**h**). *Horizontal lines* represent median values; *scale bar* 50 µm; **P < 0.01, ***P < 0.001

The vascular maturation status of PN *IDH1* ^*mut*^, CLAS-like and MES-like GBMs was then assessed by IHC analyses for the expression of the basement membrane marker ColIV (Fig. 3a) and pericyte coverage of vessels was assessed by staining for α-SMA (Fig. 3e). The mRNA expression of these markers in the Verhaak cohort [8] indicated a lower expression level of COL4A1 in PN tumors (Fig. 3b), and an increased expression level of ACTA2 in MES tumors (Fig. 3f). At protein level we observed similar patterns in the Groningen cohort by IHC analyses (Fig. 3c, g). However, as we had observed differences in MVD-values between the subclasses, we decided to correct for the endothelial lining of the vessels. The positive pixel count was therefore divided by the total vascular perimeter measurement. By doing so, the trend for lower ColIV expression in PN *IDH1* ^*mut*^ tumors was not maintained (Fig. 3d). The trend for increased α-SMA expression in MES-like tumors was still visible, but a little less clear (Fig. 3h). The comparison of hypoxic and normoxic tissue did not report differences in vascular maturation status between the subclasses (Online Resource 5).

### Angiogenic signaling pathway upregulation is not associated with GBM subclasses

Finally, to assess whether angiogenic signaling was different in molecular subclasses of GBM the expression levels of several angiogenic signaling factors was quantified. The most important angiogenic functions of the specific signaling molecules are summarized in Online Resource 6.

**Fig. 4:**
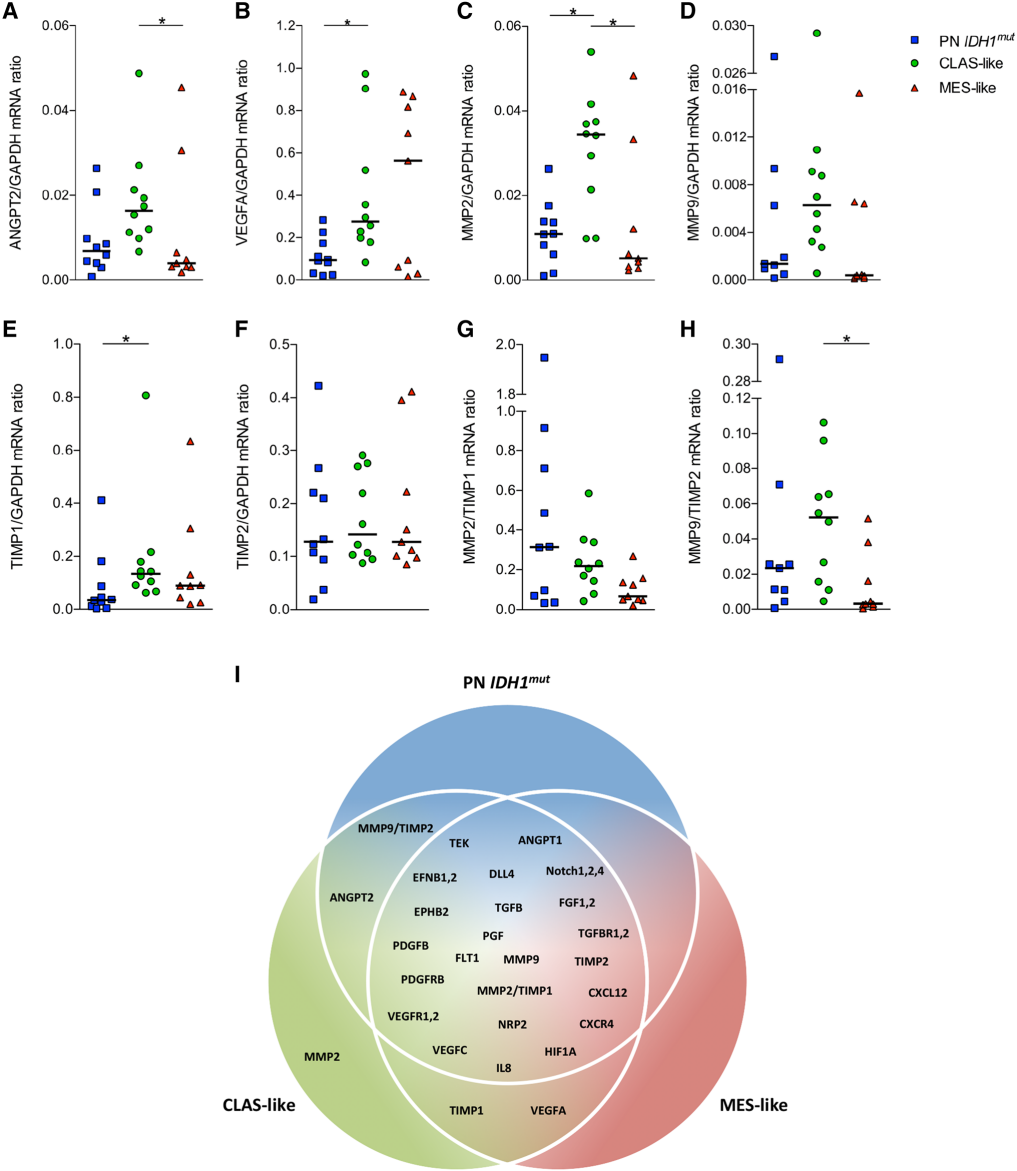
Ample angiogenic signaling factors are differentially expressed in GBM subclasses, preferably affecting the CLAS-like GBMs. Of the 31 angiogenic signaling factors that were analyzed, 4 were found to be differentially expressed between the subclasses as was evaluated by qRT-PCR. The pro-angiogenic factors ANGPT2 (**a**) and VEGFA (**b**) were found to be highly expressed in CLAS-like GBMs. VEGFA expression was also high in MES-like tumors, but with a bigger variation in expression level and therefore not significantly different. MMP2 (**c**) was upregulated in CLAS-like GBMs, but this was accompanied by upregulated expression of its endogenous inhibitor TIMP1 (**e**). MMP9 (**d**) and its inhibitor TIMP2 (**f**) were not differentially expressed in the subclasses. Dividing the expression of the tissue proteases by the expression levels of their endogenous inhibitors revealed that there was no differential expression of MMP2/TIMP1 (**g**), but MMP9/TIMP2 was actually upregulated in CLAS-like GBMs (**h**). In the Venn diagram the differential expression of angiogenic factors can be appreciated, with the majority of markers in the middle shared region, thereby reflecting the similarity in signaling patterns of the molecular subclasses (**i**). Individual mRNA ratios per tumor are displayed and *horizontal lines* represent median expression values; *P < 0.05

Out of the 31 assessed factors, only 4 were differentially expressed in the subclasses. The pro-angiogenic factors angiopoietin-2 (ANGPT2) and vascular endothelial growth factor A (VEGFA) were found to be expressed at a higher level by CLAS-like tumors (Fig. 4a, b). In addition to these pro-angiogenic factors, the expression of matrix metalloproteinase 2 (MMP2) was also elevated in CLAS-like tumors (Fig. 4c). However, its endogenous inhibitor, tissue inhibitor of metallopeptidase 1 (TIMP1), was also upregulated in CLAS-like tumors (Fig. 4e).

When the expression of the MMPs was related to the expression of their corresponding endogenous inhibitors (TIMPs), it appeared that while MMP2 was differentially expressed at an absolute level, the corrected expression ratio MMP2/TIMP1 did not differ between the molecular subtypes (Fig. 4g). On the contrary, uncorrected MMP9 was not differentially expressed (Fig. 4d), but the MMP9/TIMP2 ratio was different between the subclasses with the highest level observed for CLAS-like GBMs (Fig. 4h).

In Fig. 4i the differential expression of the assessed angiogenic targets in molecular subclasses is depicted in a Venn diagram. It can be appreciated that the molecular subclasses of GBM have highly similar expression patterns of angiogenic factors.

## Discussion

Because of the highly vascularized nature of GBMs we hypothesized that anti-angiogenic therapy could have an effect in these tumors. The novel therapeutics that seemed promising in pre-clinical studies, unfortunately, only rarely produced similar results in clinical trials. For example Bevacizumab, a promising anti-angiogenic drug in the pre-clinical setting, only resulted in an improvement of progression-free survival and left the overall survival of patients unaltered in two large Phase 3 randomized clinical trials when it was added to standard chemotherapy for newly diagnosed GBM patients [25, 26]. A slight improvement in overall survival was observed for Bevacizumab addition to chemotherapy (Lomustine) for recurrent GBM patients [27], but it is evident that more extensive research is needed to identify patients that could potentially benefit from these types of treatments. Therefore, in this study we aimed to identify vascular abnormalities specific of the PN *IDH1* ^*mut*^, CLAS-like and MES-like subclasses of GBM.

In agreement with previous reports, we found that MES-like tumors are enriched for necrosis [21]. As it is known that necrotic tissue is often surrounded by hypoxic areas [22], and hypoxia is a potent inducer of angiogenesis, high angiogenic potential was expected from MES tumors. By performing extensive characterization of the vasculature we were able to establish that MES-like tumors have larger, but not more vessels than other subclasses. This contradicted our hypotheses, since active angiogenesis would likely have resulted in predominantly small, newly formed vessels. Previous studies have however shown that larger vessels do not necessarily indicate better oxygenation, as the oxygenation status is more dependent on blood flow rate and diffusion distance from the vessel to the cells [28]. Therefore, an increased flow rate in these tumors could still have resulted in a lower oxygenation level in MES-like tumors. Reduced perfusion in the MES-like tumors thus provides a potential connection between the larger vessel size in this subclass and the increased rate of necrosis and hypoxia. In this regard it would be interesting to analyze vaso-occlusive events in GBM, which itself has a profound effect on blood flow and oxygenation of target tissues [29].

Furthermore, neo-angiogenesis could also be reflected by a more abundant expression of endothelial markers and a lower maturation rate of vessels. These hypotheses were partly confirmed by the data from the TCGA data repository, as those indicated elevated expression levels of endothelial markers CD34 and ENG in MES tumors at the mRNA level. At the protein level we were unable to identify such differences. Against our expectation, we found that the vessel maturity markers α-SMA and ColIV were elevated at mRNA level in the MES-like subclass compared to the PN *IDH1* ^*mut*^ subclass, and a similar trend was also observed at protein level. Because we established through morphometry that MES-like tumors have larger vessels we then applied a correction for vessel size. This correction led to fading of the differences observed at protein level, implying that the vessel size explained (part of) the differences in expression and there is no true difference in basement membrane deposition or pericyte coverage around the vessels of GBM subclasses. We believe that although the corrections for the number of vessels and vessel perimeter introduced differences between the mRNA and protein analyses, they can provide very useful information. It was due to these corrections that we were able to elucidate that the increased expression level of basement membrane and pericyte markers could actually be explained by an increase of vascular size.

The current study did not reveal an association between the angiogenic signaling network and GBM subclasses. As outlined earlier, genetic aberrations linked to particular molecular subclasses were presumed to affect different angiogenic downstream targets, however the expression of these targets appeared not to be different between the subclasses. Surprisingly, the few upregulated angiogenic targets were actually highest in CLAS-like and not MES-like tumors.

Given the scarceness of pre-clinical data it is interesting that a clinical trial with anti-angiogenic therapy (Bevacizumab) has included an analysis for the molecular subtypes. In this trial standard of care was supplemented with Bevacizumab (anti-VEGFA treatment) and especially the *IDH1* wild-type PN tumors benefited from the addition of Bevacizumab [10]. Since our selection of PN tumors was based on an *IDH1* mutation, we were unable to address this subset of PN tumors.

In conclusion, we report that although MES-like GBMs have larger vessels and PN *IDH1* ^*mut*^ tumors tend to possess less necrosis and hypoxia, no major differences in angiogenic signaling patterns were observed between GBM subclasses at the time of diagnosis. Although our study did not functionally address neo-angiogenesis, by the use of vascularization patterns and signaling signatures as a measure for the outcome of neo-angiogenic processes, our findings challenge the concept of MES GBMs being more angiogenic. It can be presumed that the lack of correction in prior studies possibly has led to the erroneous expectation of increased angiogenic activity in the MES subclass. As such, our results do not support attempts to differentiate anti-angiogenic treatment according to the GBM molecular subtypes assessed in this study.

## Electronic supplementary material

Below is the link to the electronic supplementary material.

Supplementary material 1 (DOCX 29 KB)

The probability of overall survival for MES-like GBMs is significantly worse than for other GBM subclasses. Kaplan-Meier analysis for molecular subclasses of GBM confirms the previously described worse survival pattern for MES-like GBMs compared to other subclasses. Statistical evaluation was performed by the Log-rank test (*P* < 0.05). (TIF 1721 KB)

Separation of normoxic and hypoxic tumor areas based on CAIX expression. The quantification of CAIX as a selection marker for normoxic and hypoxic tissue areas illustrates enrichment of CAIX expression in hypoxic tissue areas in all subclasses. *Horizontal lines* represent median scores of the groups; ***: *P* < 0.001. (TIF 561 KB)

Endothelial marker expression and MVD values in normoxic and hypoxic tumor areas. Endothelial markers CD34 (**A**) and ENG (**B**) were expressed at similar levels in all subclasses in both normoxic and hypoxic tumor areas. The number of vessels per mm^2^ is increased in hypoxic areas in CLAS-like GBMs, but not in the other subclasses (**C**). The vessel area is largest in MES-like tumors but only significantly larger in normoxic areas (**D**), whereas the vessel perimeter in MES-like tumors is increased in both normoxic and hypoxic tumor areas (**E**). *Horizontal lines* represent median scores of the groups; *: *P* < 0.05, **: *P* < 0.01. (TIF 5067 KB)

The vascular maturation status is similar in normoxic and hypoxic tumor areas. No differences and comparable patterns were observed in normoxic and hypoxic tumor areas for the ColIV positive pixel fraction (**A**), ColIV layer thickness (**B**), α-SMA positive pixel fraction (**C**) and α-SMA layer thickness (**D**). *Horizontal lines* represent median scores of the groups. (TIF 3639 KB)

Summary of the factors assessed as representatives of different angiogenic signaling pathways. (DOCX 17 KB)
