# Supplementary material for: Novel insights into vascularization patterns and angiogenic factors in glioblastoma subclasses
Source: J Neurooncol. 2016 Sep 15;131(1):11–20. doi: 10.1007/s11060-016-2269-8 (PMC5258811; doi:10.1007/s11060-016-2269-8)
Supplement: Supplementary file 1 — Supplementary material 1 (DOCX 29 KB) [file 11060_2016_2269_MOESM1_ESM.docx]

**Online Resource 1. Supplementary Methods**

***Patient population***

PN samples harbored the *IDH1^R132H^* mutation, CLAS samples had *EGFR* amplification combined with EGFR and EGFRvIII (over)expression, whereas MES tumors had high expression of at least 2 important MES markers (phosphatase and tensin homolog [PTEN], vimentin [VIM] and/or chitinase 3-like 1 [YKL40]). The samples in the current study represent samples that were described previously and other *IDH1^R132H^* mutant GBM samples [1].

The data from the Verhaak cohort were generated through the Genechip Human Genome HT-HG-U133A platform [2].

***Detailed IHC procedure***

Sections of 4 µm-thickness were cut from FFPE tissue in series and these were used for detection of expression of Carbonic anhydrase IX (CAIX), CD34, Endoglin (ENG), collagen type IV alpha 1 (ColIV) and α-smooth muscle actin (α-SMA). The tissue sections were deparaffinized in xylol and rehydrated in graded ethanol series. The staining for CAIX did not require an antigen retrieval step, but for CD34, Col IV and ENG IHC-staining heat-induced antigen retrieval was performed for 15 minutes in Tris-HCl/EDTA buffer pH 9.0, EDTA buffer pH 8.0 and Tris/HCl pH 9.0, respectively. The staining for α-SMA required incubation of the slides overnight in Tris/HCl buffer pH 9.0 at 80ºC. Endogenous peroxidase was blocked for 30 minutes using 0.3% H_2_O_2_. Then the tissue sections were incubated with mouse anti-CAIX (1:50, Clone M75, Siemens Medical Diagnostics, Erlangen, Germany), mouse anti-CD34 primary antibody (ready-to use, Clone QBend10, Beckman Coulter, Marseille, France), mouse anti-ENG (1:1000, Clone 3A9, Novus Biologicals, Littleton, CO), rabbit anti-ColIV (1:100, MP Biomedicals, Santa Ana, CA) or mouse anti-α-SMA (1:800, Clone 1A4, DAKO, Glostrup, Denmark) for 1 hour at room temperature. For detection of CAIX, ColIV, ENG and α-SMA sections were incubated with appropriate secondary and tertiary HRP-conjugated antibodies (DAKO, 1:100), both for 30 minutes 1:100 diluted in 1% BSA in PBS with the addition of 1% AB-serum. For CD34-detection sections were incubated with alkaline phosphatase-conjugated goat anti-mouse antibody (DAKO, 1:500) for 30 minutes. The immunoreactivity of CAIX, ColIV, ENG and α-SMA was visualized using 3,3-diaminobenzidine (DAB) solution, and CD34-staining was visualized by Fast Red TR/Naphthol AS-MX-phosphate (Sigma-Aldrich, St. Louis, MO). Cell nuclei were counterstained using hematoxylin for all types of stainings.

***Morphometrical analyses***

Tissue sections stained for CD34 were assessed in 5 hot-spot fields at 200x magnification (0.196 µm^2^) [3-5]. To quantify the average vessel area and vessel perimeter digital scans of the IHC-stainings for CD34, ENG, ColIV and α-SMA were obtained by scanning the slides with a C9600 NanoZoomer (Hamamatsu Photonics KK, Hamamatsu City, Japan). All computer-assisted morphometry was subsequently performed on these scans using Aperio ImageScope software version 12.1.0 (Leica Microsystems, Vista, CA). Vessels with a diameter of minimally 5 µm were manually delineated on both the scans of ColIV and α-SMA stained tissue sections for quantification of the vessel area and vessel perimeter.

***Histological evaluation***

The expression level of hypoxia marker CA-IX, endothelial markers CD34 and ENG, basement membrane marker ColIV and pericyte marker α-SMA were quantified using Aperio ImageScope software. Vital tumor tissue was manually delineated for every individual section and staining, and hypoxic tissue was delineated using the CAIX staining pattern as guidance. False positive staining in vessel lumina due to the presence of erythrocytes was excluded from analyses.

Positive staining for α-SMA and ColIV was primarily observed at the surrounding of vessels. First of all, the positive pixel percentage was calculated by division of the surface area found positive (in pixels) by the total area of the vital, normoxic or hypoxic field (in pixels). Secondly, since staining for α-SMA and ColIV was specifically identified around vessels, we also attempted to obtain an indication of the thickness of these layers by dividing the number of positive pixels by the total vessel perimeter of that specific tissue sample.

CD34 and ENG showed clear vessel-associated expression, but both of them also stained areas where no clear vessel lumen could be observed, i.e. areas that could well be interpreted as sites of neo-angiogenesis. Therefore, these stainings were analyzed only for positive pixel percentage.

***Microfluidic cards***

RNA was purified from 30 snap-frozen GBM biopsy samples using TRIzol reagent (Invitrogen, Karlsruhe, Germany). RNA was precipitated with isopropanol and the air-dried pellet was resuspended in RNase-free water. RNA content was quantified using Nanodrop ND-1000 IV spectrophotometer. Up to 1 µg RNA was reverse transcribed using Superscript II reverse transcriptase (Invitrogen, Karlsruhe, Germany) and random hexamer primers (Promega, Leiden, the Netherlands). Successful cDNA synthesis was confirmed through PCR for GAPDH. Quantitative gene expression analyses were obtained with Taqman assays which were preloaded onto custom-designed Taqman array Micro Fluidic Cards (low-density array, Applied Biosystems, Foster City, CA). The custom-designed arrays contained 8 sample fill reservoirs, each with 16 detectors in triplicate (48 assays per fill reservoir). The qRT-PCR was performed in triplicate with the Taqman® Universal PCR Master Mix (without AmpErase® UNG, Applied Biosystems), using cDNA at concentration 2.5 ng/µl (100 µl per fill reservoir) on an ABI ViiA^TM^ 7 real-time sequence detection system (Applied Biosystems).

Assays were included for 31 genes of interest and one endogenous control (see table below), implying that two consecutive slots were required to be loaded with the same sample to obtain all measurements for one sample. The following settings were used for qRT-PCR: 50ºC for 2 minutes and initial denaturation at 95ºC for 10 minutes, followed by 40 cycles of amplification at 95ºC for 15 seconds and 60ºC for 10 minutes. Gene expression for each assay was measured as fold change in amplication relative to a reference gene. Data was analyzed using the comparative C_t_ method with ViiA^TM^ software (v1.2.4, Applied Biosystems).

**Microfluidic card details**

| Gene symbol | Gene name | Assay ID |
| --- | --- | --- |
| ANGPT1 | Angiopoietin 1 | Hs00375822_m1 |
| ANGPT2 | Angiopoietin 2 | Hs01048042_m1 |
| CXCL12 | Chemokine (C-X-C motif) ligand 12 | Hs00171022_m1 |
| CXCR4 | Chemokine (C-X-C motif) receptor 4 | Hs00237052_m1 |
| DLL4 | Delta-like 4 (Drosophila) | Hs00184092_m1 |
| EFNB1 | Ephrin-B1 | Hs00270004_m1 |
| EFNB2 | Ephrin-B2 | Hs00187950_m1 |
| EPHB2 | EPH receptor B2 | Hs00362096_m1 |
| FGF1 | Fibroblast growth factor 1 | Hs01092738_m1 |
| FGF2 | Fibroblast growth factor 2 | Hs00266645_m1 |
| FLT1 | Fms-related tyrosine kinase 1 | Hs01052961_m1 |
| GAPDH | Glyceraldehyde-2-phosphate dehydrogenase | Hs99999905_m1 |
| HIF1A | Hypoxia inducible factor 1, alpha subunit | Hs00153153_m1 |
| IL8 | Interleukin 8 | Hs00174103_m1 |
| KDR | Kinase insert domain receptor (a type III receptor tyrosine kinase | Hs00911700_m1 |
| MMP2 | Matrix metalloproteinase 2 | Hs01548727_m1 |
| MMP9 | Matrix metalloproteinase 9 | Hs00234579_m1 |
| NOTCH1 | Notch 1 | Hs01062014_m1 |
| NOCTH2 | Notch 2 | Hs01050702_m1 |
| NOTCH4 | Notch 4 | Hs00965889_m1 |
| NRP2 | Neuropilin 2 | Hs00187290_m1 |
| PDGFB | Platelet-derived growth factor beta polypeptide | Hs00966522_m1 |
| PDGFRB | Platelet-derived growth factor receptor, beta polypeptide | Hs01019589_m1 |
| PGF | Placental growth factor | Hs00182176_m1 |
| TEK | TEK tyrosine kinase, endothelial | Hs00945146_m1 |
| TGFB1 | Transforming growth factor, beta 1 | Hs00998133_m1 |
| TGFBR1 | Transforming growth factor, beta receptor 1 | Hs00610320_m1 |
| TGFBR2 | Transforming growth factor, beta receptor II | Hs00234253_m1 |
| TIMP1 | TIMP metallopeptidase inhibitor 1 | Hs00171558_m1 |
| TIMP2 | TIMP metallopeptidase inhibitor 2 | Hs00234278_m1 |
| VEGFA | Vascular endothelial growth factor A | Hs00900055_m1 |
| VEGFC | Vascular endothelial growth factor C | Hs00153458_m1 |

**References**

1. Conroy S, Kruyt FA, Joseph JV, Balasubramaniyan V, Bhat KP, Wagemakers M, et al. Subclassification of Newly Diagnosed Glioblastomas through an Immunohistochemical Approach. PLoS One. 2014;9: e115687.

2. Verhaak RG, Hoadley KA, Purdom E, Wang V, Qi Y, Wilkerson MD, et al. Integrated genomic analysis identifies clinically relevant subtypes of glioblastoma characterized by abnormalities in PDGFRA, IDH1, EGFR, and NF1. Cancer Cell. 2010;17: 98-110.

3. Brem S, Cotran R, Folkman J. Tumor angiogenesis: a quantitative method for histologic grading. J Natl Cancer Inst. 1972;48: 347-356.

4. Vermeulen PB, Gasparini G, Fox SB, Colpaert C, Marson LP, Gion M, et al. Second international consensus on the methodology and criteria of evaluation of angiogenesis quantification in solid human tumours. Eur J Cancer. 2002;38: 1564-1579.

5. Sie M, de Bont ES, Scherpen FJ, Hoving EW, den Dunnen WF. Tumour vasculature and angiogenic profile of paediatric pilocytic astrocytoma; is it much different from glioblastoma? Neuropathol Appl Neurobiol. 2010;36: 636-647.
