# Supplementary figures and images for: Novel insights into vascularization patterns and angiogenic factors in glioblastoma subclasses
Source: J Neurooncol. 2016 Sep 15;131(1):11–20. doi: 10.1007/s11060-016-2269-8 (PMC5258811; doi:10.1007/s11060-016-2269-8)

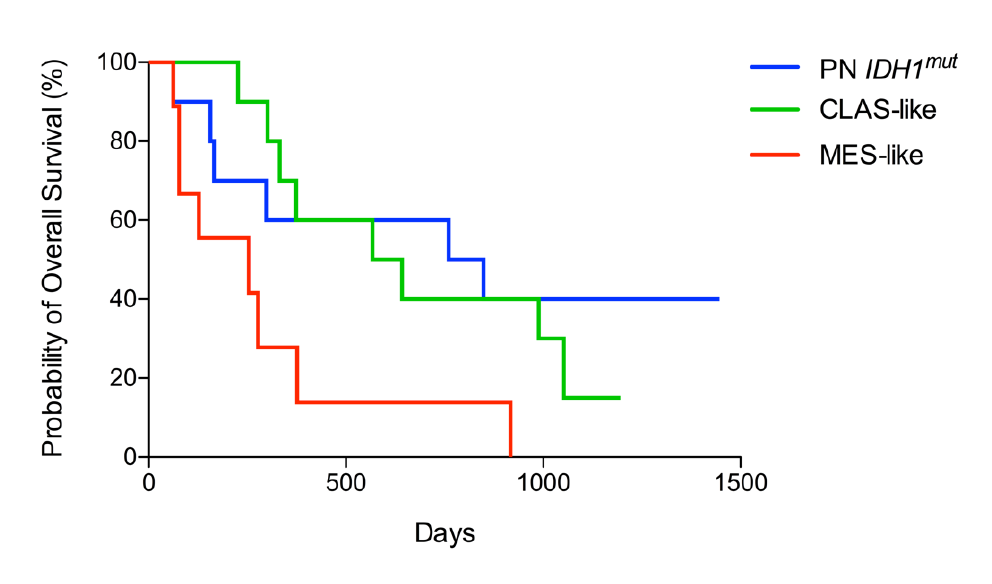

Supplement: Supplementary file 2 — The probability of overall survival for MES-like GBMs is significantly worse than for other GBM subclasses. Kaplan-Meier analysis for molecular subclasses of GBM confirms the previously described worse survival pattern for MES-like GBMs compared to other subclasses. Statistical evaluation was performed by the Log-rank test (P < 0.05). (TIF 1721 KB) [file 11060_2016_2269_MOESM2_ESM.tif]

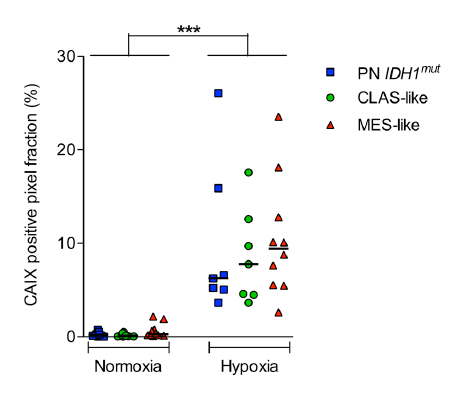

Supplement: Supplementary file 3 — Separation of normoxic and hypoxic tumor areas based on CAIX expression. The quantification of CAIX as a selection marker for normoxic and hypoxic tissue areas illustrates enrichment of CAIX expression in hypoxic tissue areas in all subclasses. Horizontal lines represent median scores of the groups; ***: P < 0.001. (TIF 561 KB) [file 11060_2016_2269_MOESM3_ESM.tif]

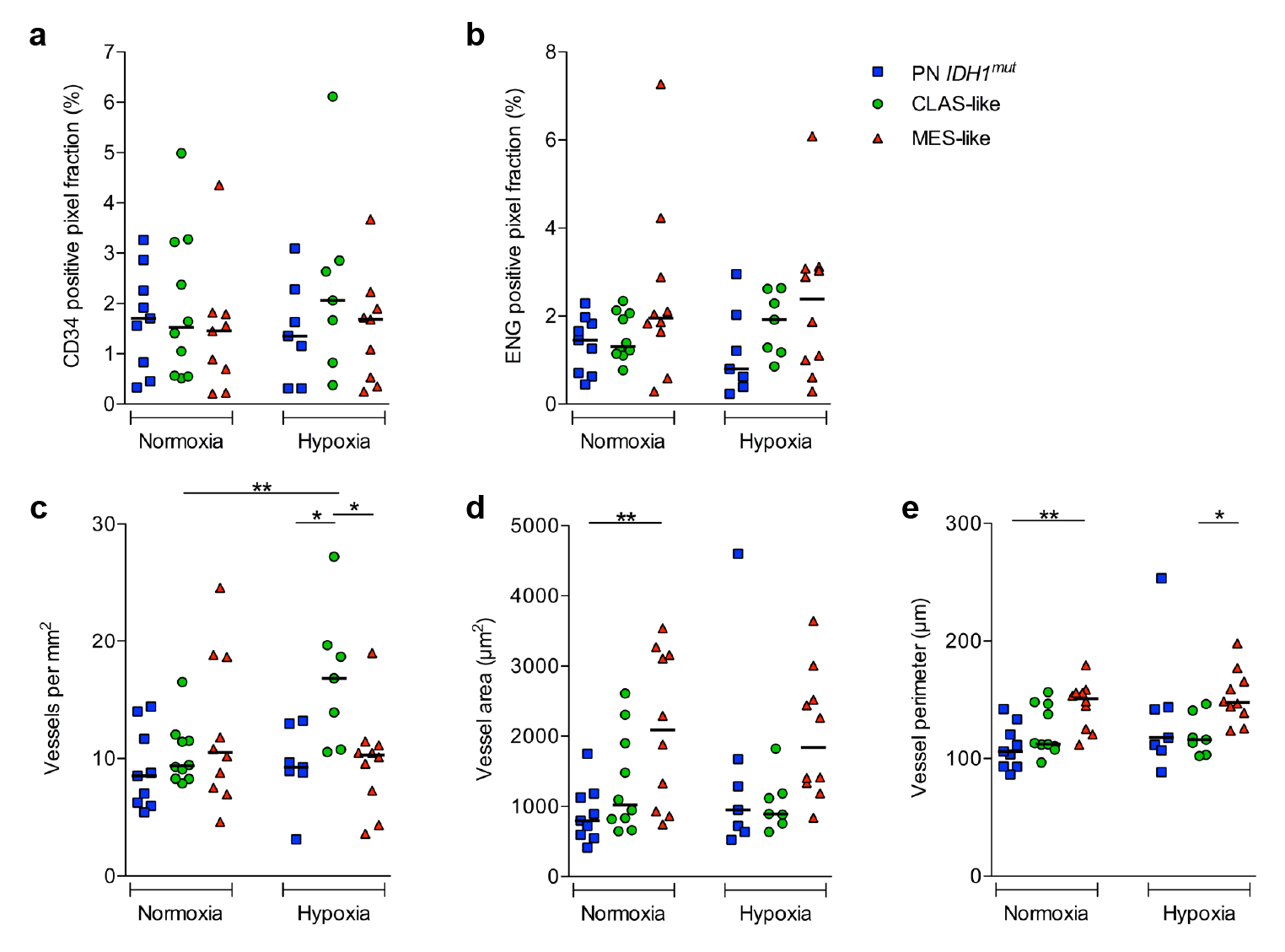

Supplement: Supplementary file 4 — Endothelial marker expression and MVD values in normoxic and hypoxic tumor areas. Endothelial markers CD34 (A) and ENG (B) were expressed at similar levels in all subclasses in both normoxic and hypoxic tumor areas. The number of vessels per mm2 is increased in hypoxic areas in CLAS-like GBMs, but not in the other subclasses (C). The vessel area is largest in MES-like tumors but only significantly larger in normoxic areas (D), whereas the vessel perimeter in MES-like tumors is increased in both normoxic and hypoxic tumor areas (E). Horizontal lines represent median scores of the groups; *: P < 0.05, **: P < 0.01. (TIF 5067 KB) [file 11060_2016_2269_MOESM4_ESM.tif]

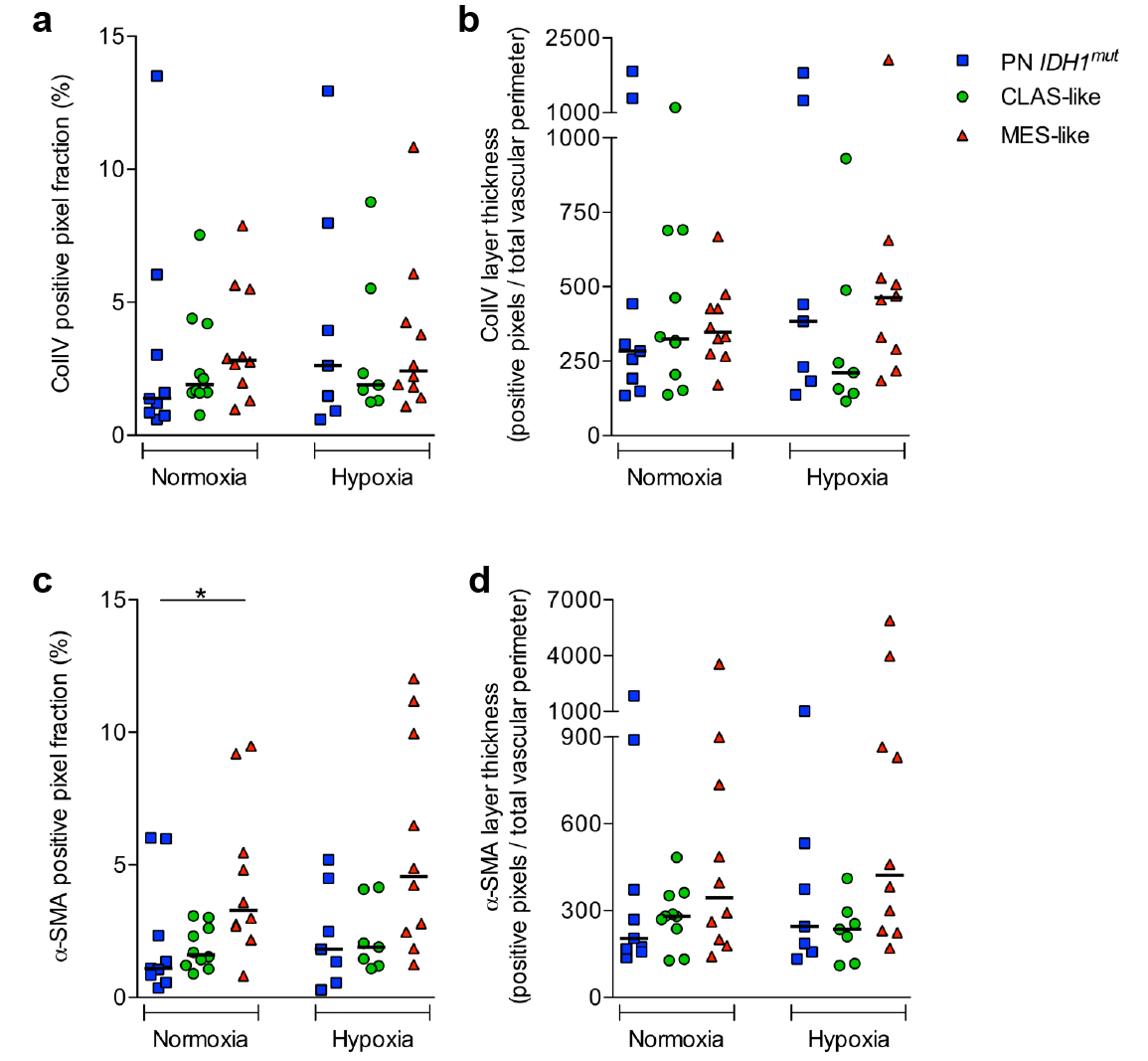

Supplement: Supplementary file 5 — The vascular maturation status is similar in normoxic and hypoxic tumor areas. No differences and comparable patterns were observed in normoxic and hypoxic tumor areas for the ColIV positive pixel fraction (A), ColIV layer thickness (B), α-SMA positive pixel fraction (C) and α-SMA layer thickness (D). Horizontal lines represent median scores of the groups. (TIF 3639 KB) [file 11060_2016_2269_MOESM5_ESM.tif]
