# Supplementary material for: Novel insights into vascularization patterns and angiogenic factors in glioblastoma subclasses
Source: J Neurooncol. 2016 Sep 15;131(1):11–20. doi: 10.1007/s11060-016-2269-8 (PMC5258811; doi:10.1007/s11060-016-2269-8)
Supplement: Supplementary file 6 — Summary of the factors assessed as representatives of different angiogenic signaling pathways. (DOCX 17 KB) [file 11060_2016_2269_MOESM6_ESM.docx]

**Online Resource 6. Summary of the factors assessed as representatives of different angiogenic signaling pathways**

| Gene | Putative role in angiogenesis |
| --- | --- |
| *Angiopoietin-TIE-2 pathway* |  |
| ANGPT1 (Ang1) | Vessel stabilization / maturation, tightening of the EC-barrier, vessel growth stimulation under hypoxic conditions |
| ANGPT2 (Ang2) | VEGF-dependent vessel destabilization, pericyte detachment and sensitization of endothelial cells to proangiogenic signaling |
| TEK (TIE2) | Receptor for Ang-1 and Ang-2 |
|  |  |
| *Delta-notch pathway* |  |
| DLL4 | Signaling of tip cells in angiogenic sprouting |
| NOTCH1/2/4 | Receptors for DLL4, activation of Notch decreases VEGFR2 and VEGFR3 expression |
|  |  |
| *Ephrins* |  |
| EFNB1 | EC-migration and attachment |
| EFNB2 | EC-migration and attachment; mediation of interaction between ECs and vascular smooth muscle cells |
| EPHB2 | Receptor for Ephrin B-type ligands |
|  |  |
| *Growth factor signaling* |  |
| FGF1 | EC migration, proliferation and tube formation |
| FGF2 (bFGF) | EC migration, proliferation and tube formation |
| PGF | Recruitment and activation of various cell types that up-regulate pro-angiogenic factors |
| PDGFB | Pericyte maturation and induction of VEGF |
| PDGFRB | Receptor for PDGFB |
| TGFB1 | Induction of other angiogenic factors (e.g. FGF-2, PDGF and PDGFR) |
| TGFBR1 | Receptor for TGFB |
| TGFBR2 | Receptor for TGFB |
|  |  |
| *Tissue remodeling factors* | |
| MMP2/9 | Degradation of extracellular matrix proteins to facilitate angiogenesis |
| TIMP1 | Endogenous inhibitor of MMP2 |
| TIMP2 | Endogenous inhibitor of MMP9 |
|  |  |
| *VEGF pathway* |  |
| FLT1 (VEGFR1) | Receptor for VEGFC and PGF; decoy receptor for KDR |
| KDR (FLK-1, VEGFR2) | Receptor for VEGFA, plays a role in endothelial cell maturation |
| NRP2 | Receptor for VEGFA and VEGFC; interaction between NRP2 and KDR upon VEGF-binding |
| VEGFA | Induction of sprouting, EC proliferation, and a pleiotropy of other proangiogenic factors |
| VEGFC | Induction of sprouting and vessel permeability |
|  |  |
| *Other angiogenic signaling factors* | |
| CXCL12 (SDF-1) | EC migration; hypoxia activates CXCL12-CXCR4 axis |
| CXCR4 | Receptor for CXCL12; induction of CXCR4 by VEGF |
| HIF1A | Proangiogenic signaling factor; induction of multiple angiogenic signaling pathways |
| IL8 (CXCL8) | EC migration, proliferation and tube formation; expressed under hypoxic conditions; increases MMP2 and MMP9 expression |
